# Morphological analysis of isolated hemivertebra: radiographic manifestations related to the severity of congenital scoliosis

**DOI:** 10.1186/s12891-024-07193-8

**Published:** 2024-02-05

**Authors:** Tianhua Rong, Yang Jiao, Yizhen Huang, Erwei Feng, Heng Sun, Junduo Zhao, Jianxiong Shen

**Affiliations:** Department of Orthopedics, Peking Union Medical College Hospital, Peking Union Medical College, Chinese Academy of Medical Science, Beijing, People’s Republic of China

**Keywords:** Isolated hemivertebra, Congenital scoliosis, Morphological analysis, Progression, Observation

## Abstract

**Purpose:**

The natural history of congenital scoliosis (CS) caused by hemivertebra varies greatly. This study aimed to explore the association between the morphology of hemivertebra and the severity of CS, since the diagnosis of the hemivertebra.

**Methods:**

Patients with isolated (single fully segmented) hemivertebra were enrolled. The degree and progression of deformity were compared by three morphological parameters of hemivertebra, comprising whether the width of hemivertebra extends across the central vertical line of lower adjacent vertebra (midline); the lateral height ratio (LHR, lateral height of hemivertebra× 2/(lateral height of HV-1 plus HV + 1) with the cut-point being 0.9; and the sagittal position of hemivertebra that was divided into the lateral and posterolateral group.

**Results:**

In total, 156 patients (mean age 9.7 ± 6.2 years, 81 males) were enrolled. The number of thoracic, thoracolumbar (T12/13-L1), and lumbar hemivertebrae were 63, 41, and 52, respectively. Hemivertebrae across the midline had larger scoliosis and kyphosis (58.3 ± 20.6° vs. 42.8 ± 15.0°, *P* <  0.001; 45.1 ± 32.5° vs. 29.5 ± 25.7°, *P* = 0.013, respectively). Hemivertebrae with LHR ≥0.9 was associated with larger scoliosis (55.7 ± 20.6° vs. 41.4 ± 13.3°, *P* <  0.001). Larger scoliosis and kyphosis were observed in posterolateral hemivertebrae (54.4 ± 21.0° vs. 44.4 ± 15.6°, *P* = 0.026; 51.4 ± 31.5° vs. 20.6 ± 17.1°, *P* <  0.001, respectively). Co-occurrence of more than one of the three positive parameters above indicated higher annual progression (5.0 ± 2.2° vs. 3.3 ± 1.3°, *P* <  0.001).

**Conclusion:**

Three positive parameters, width across the midline, LHR ≥0.9, and posterolateral position were associated with a more severe deformity in patients with isolated hemivertebra. Hemivertebrae with more than one positive parameter may cause progressive deformity, and thus need prompt surgery.

**Level of evidence:**

Prognostic, level IV.

## Introduction

Vertebral malformation causes asymmetric spinal growth and results in congenital scoliosis (CS). CS is classified into three types: failure of formation, failure of segmentation, and mixed type [1, 2]. As the most prevalent subtype of failure of formation, a hemivertebra (HV) is a irregular-shaped structure, which typically consists of half a vertebral body, a single pedicle, and hemilamina [3]. McMaster and David further divided HVs into three types: fully-segmented, semi-segmented and unsegmented [4]. The progression of HV-induced CS varies greatly from 1° to 33° per year, and the relevant factors include the location, number and type of HV [4, 5]. In single HV, the fully-segmented type is most likely to produce progressive deformity, and prophylactic operation may be needed [4, 6]. Resection with instrumentation is reported to be an effective procedure in dealing with HV [7–12]. A trend of early HV resection in juvenile or even infantile patients with HV-induced CS has been reported recently [13–15].

**Fig. 1:**
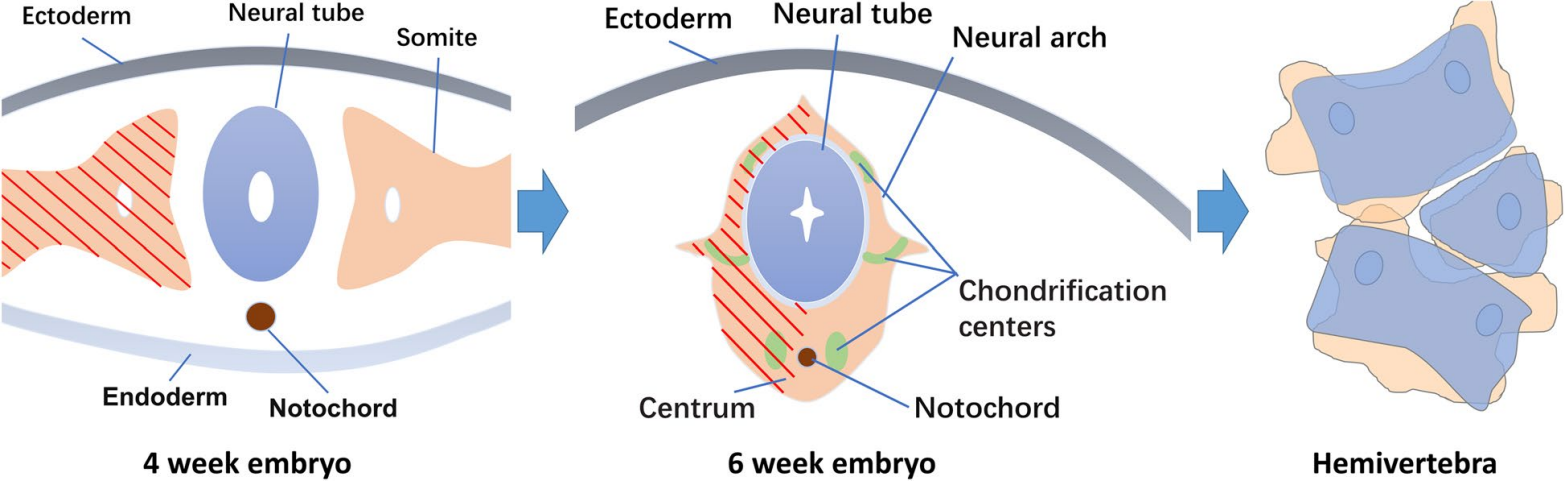
Sketch map of the pathogenesis of hemivertebra from the perspective of embryonic development. The red oblique lines represent the unilateral dysplasia of somite

In decision-making, it is important to refrain from excessive treatment and to notice that not all HVs need prompt resection. Studies have shown that in some patients, the deformity caused by single HV can remain steady [4, 16]. Winter et al. reported seven patients with spontaneous improvement of HV-induced CS during a 9-year follow-up [17]. In such less progressive cases, a close observation to estimate the risk of progression is reasonable. An HV results from unilateral dysplasia of somite at an early fetal stage (Fig. 1) [18, 19]. Different levels of development in the remaining half of the vertebral body lead to heterogeneities in the severity of scoliosis. From this embryological perspective, certain geometrical parameters of HV may be associated with deformity progression.

Based on past clinical experience, the three-dimensional diameter parameters of HV were determined, including the transverse diameter of the vertebral body, the relative position of lateral height ratio (LHR), and the sagittal position. The transverse diameter is related to the lateral growth of HV, while the LHR is related to the longitudinal growth, and the relative position in the sagittal position reflects the forward (ventral) growth of HV. According to the distribution of HV three-dimensional diameter parameters in the included patient population, positive indicators were set as transverse diameter crossing the midline, lateral posterior HV, and LHR ≥ 0.9, all of which were associated with more severe deformities, and the statistical strength of the association decreased sequentially. In this study, data on patients with CS were collected and analyzed. Patients were followed up after being diagnosed with CS. The morphological parameters of HV were given special attention, including the width of hemivertebra extends, the lateral height ratio and the sagittal position of HV.

This study aimed to explore the association between the morphology of isolated HVs (single, fully segmented HV) and the magnitude of deformity and identify risk factors for progression.

## Materials and methods

### Participants

With approval from the Institutional Review Board of Peking Union Medical College Hospital (protocol number, S-K1239), a prospectively collected clinical database was screened retrospectively. The inclusion criteria were as follows:

1. Patients with a definitive diagnosis of CS who were managed by the senior author (J.S.).
2. Major curve caused by isolated HV, which was defined as single, fully segmented HV without contralateral bar and fused ribs.
3. Complete imaging data including all-spine radiograph, computed tomography (CT) with three-dimensional (3-D) reconstruction, and magnetic resonance imaging (MRI).

Patients with multiple HVs; and vertebral malformation caused by an infection, tumor, or trauma were excluded.

Detailed observation and recording of the types and quantities of structural abnormalities and segmentation defects in the posterior region of HV through CT, and analysis and induction of the corresponding relationship between the anterior vertebral body and the posterior vertebral plate to construct a refined classification. Observing the intraspinal situation of CS patients caused by single HV through MRI, recording the types, quantities, and locations of intraspinal abnormalities. During the follow-up period, full spine anteroposterior lateral X-rays were widely used to evaluate disease progression.

### Morphological definition

Each normal vertebra was identified as either thoracic or lumbar and numbered sequentially from T1 to L5/6. Anatomical variation in the junctional area, including cervical rib, absent rib of T12, number variation of vertebra (T13, L6), and lumbosacral transitional vertebra, were noticed to ensure consistency in identifying vertebral levels. When the total number of normal vertebrae was less than 12 in thoracic area and/or 5 in lumbar area, the HV was defined as “intrinsic” and numbered by the corresponding level. Otherwise, the HV was regarded as an “excess” segment and numbered by intervertebral space like T12-L1. The ranges of thoracic, thoracolumbar, and lumbar HV were T1 to T11–12, T12 to L1, and L1–2 to L5/6-S1, respectively.

The anteroposterior discordance (APD) of HV was defined as the mismatch between the vertebral body and the posterior structure, which was identified on 3-D CT [20]. The intraspinal anomalies (ISA) in this study included syringomyelia, tethered cord, diastematomyelia, Chiari malformation, and other occupying lesions, which were confirmed by MRI. Correspondingly, the extraspinal anomalies (ESA) were defined as congenital malformations in organs other than the spine and spinal cord [21].

### Measurement of hemivertebra

The Cobb angle of scoliosis and kyphosis, apical vertebral translation (AVT), trunk shift (TS), and the sagittal vertical axis (SVA) were measured on radiographs, according to the definitions from the Scoliosis Research Society [22].

**Fig. 2:**
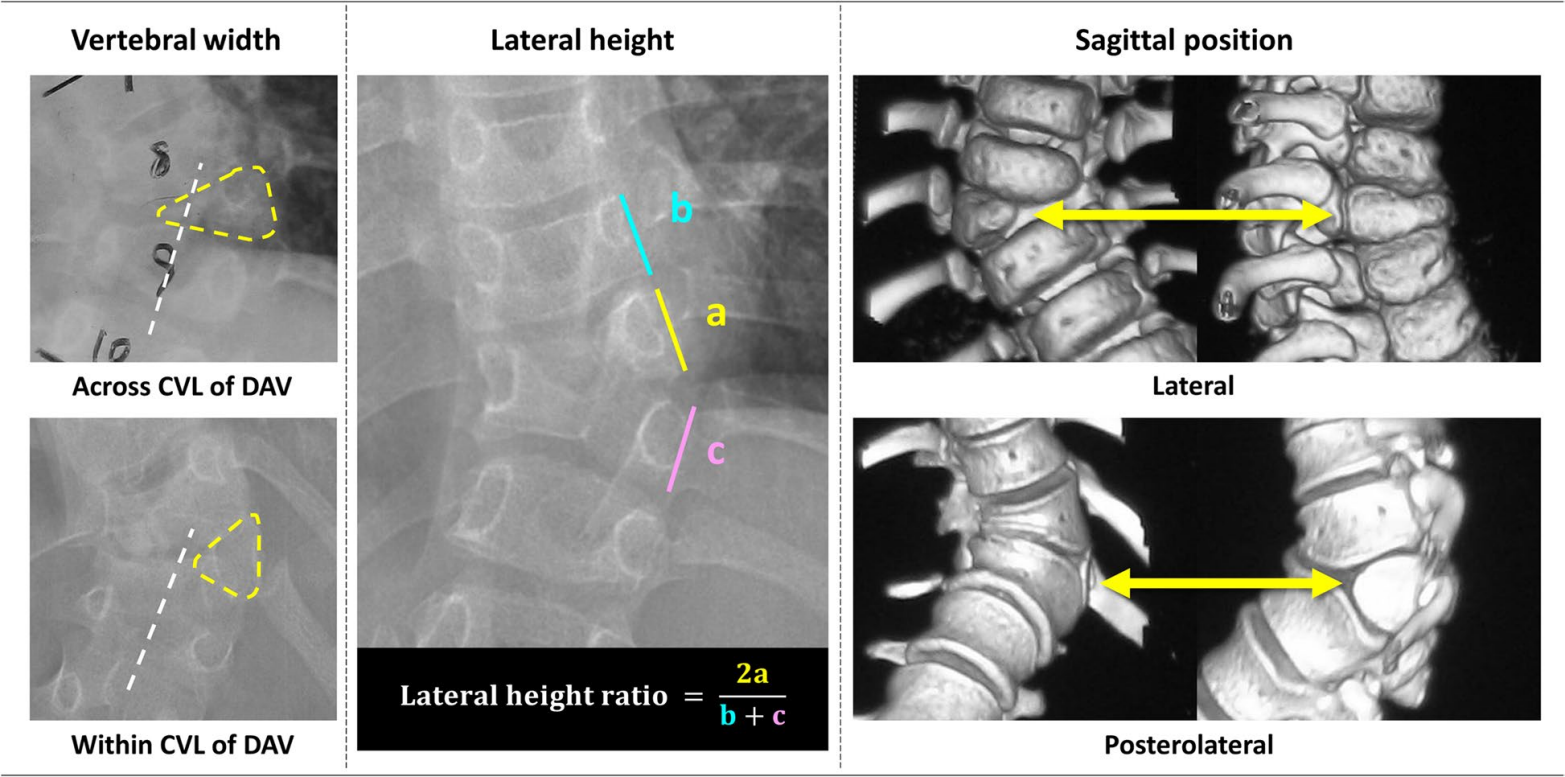
Diagram of the measurements of HV parameters. Left, HV was categorized into two groups according to whether the width extend across the “midline”, meaning the central vertical line (CVL) of distal adjacent vertebra (DAV). Middle, lateral height around HV (from HV-1 to HV + 1) was measured on convex side. The lateral height ratio (LHR) was defined as illustrated to assess the longitudinal development of HV. Right, sagittal position was divided into lateral and posterolateral group according to whether the HV extended ventrally to the anterior half of vertebral column

On coronal plane, HVs were categorized into two groups, according to whether the width extended across the “midline”, meaning the central vertical line of the distal adjacent vertebra (Fig. 2). When the HV touched the midline, it was also assigned to the “across” group. Lateral height around the HV (from HV-1 to HV + 1) was measured on the convex side. The lateral height ratio (LHR) was defined to assess the longitudinal development of HV. The calculation was twice the convex lateral height of HV divided by the summation of the proximal and distal adjacent vertebra. The grouping cut point was 0.9 (Fig. 2). The sagittal position of the HV was divided into lateral and posterolateral groups, according to whether the HV extended ventrally to the anterior half of the vertebral body (Fig. 2).

The Surgimap version 2.3 (Nemaris, NY, USA) was used for measurement [23]. Three qualified surgeons (T.R., Y.L., and H.T.) separately performed the measurement, and the average was defined as the observed value. Any inter-observer divergence of categorical variables was resolved by a consulting senior author (J.S.).

### Statistical analysis

Statistical analysis was performed using SPSS software version 22.0 (IBM, NY, USA). Student’s t-test, Mann-Whitney U test, chi-square test, one-way analyses of variance and Kruskal-Wallis test were applied for intergroup comparisons as appropriate. Spearman’s coefficient was calculated to explore the correlation between patient age and curve morphology. Analysis of covariance (ANCOVA) was performed to adjust for the influence of age when necessary. All statistical tests were 2-tailed, and a *P*-value < 0.05 was considered statistically significant.

## Results

Comparisons of clinical manifestations in patients with congenital scoliosis caused by isolated hemivertebra.

**Fig. 3:**
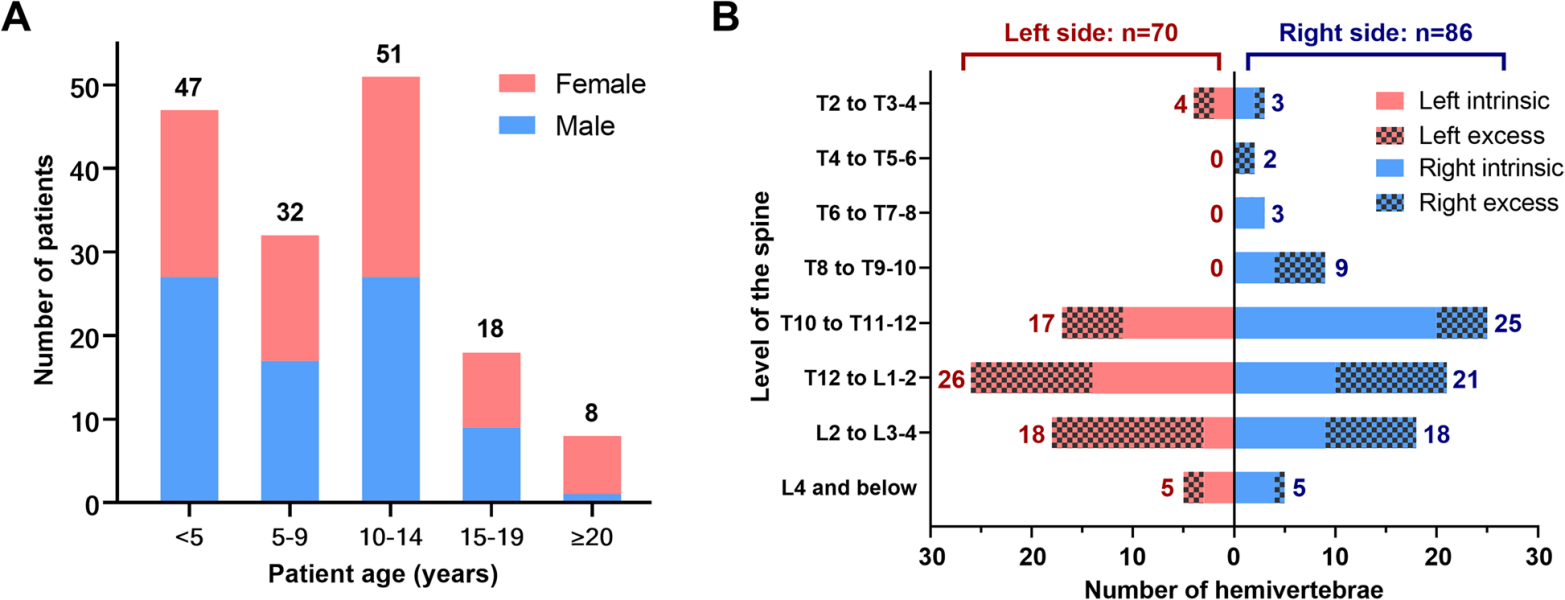
**A** Distribution of the age at diagnosis in patients with CS caused by isolated HV. **B** Distribution of side and location of enrolled hemivertebrae. Intrinsic HV indicated that the total number of vertebrae including the HV was normal (equal to or less than 12 thoracic and 5 lumbar vertebrae) and the HV was numbered by the corresponding vertebral level, e.g. T12; Excess HV indicated that the HV was a supernumerary segment in addition to the 17 or more normally developed thoracic and lumbar vertebrae, and thus was numbered by the corresponding intervertebral space, e.g. T12-L1

A total of 156 patients (81 males and 75 females) who came to our hospital between 2006 and 2019 were enrolled. The mean age at diagnosis was 9.7 ± 6.2 years. A double-peak distribution was observed with 2–5 years and 10–14 years being the most prevalent ages (Fig. 3). The average Cobb angle of scoliosis and kyphosis were 49.4° ± 19.1° and 36.2° ± 29.7°, respectively. ISA was diagnosed in 36 patients (23.1%), of which the three most common anomalies were syringomyelia in 16, tethered cord in 14, and split cord malformation in 10 cases, respectively (Table 1). Eighteen patients (11.5%) had ESA, comprising congenital heart disease in seven, renal anomalies in five, facial deformity (ptosis and/or microtia) in four, and abdominal hernia and retrosternal thyroid in one case each.

**Table 1:** Comparisons of clinical manifestations in patients with congenital scoliosis caused by isolated hemivertebra

Variables	Age		*P*-value	Side of HV		*P*-value^*^	Numbering of HV^a^		*P*-value^*^
	< 10 years (*n* = 79)	≥ 10 years (*n* = 77)		Left (*n* = 70)	Right (*n* = 86)		Intrinsic (*n* = 85)	Excess (*n* = 71)	
**Patient age (years)**	N/A	N/A	N/A	10.7 ± 7.2	8.8 ± 5.2	0.101	10.5 ± 6.9	8.6 ± 5.2	0.099
**Deformity parameters**									
**Cobb angle of scoliosis (°)**	43.7 ± 13.8	55.3 ± 22.0	< 0.001	51.8 ± 21.9	47.5 ± 16.4	0.441	52.9 ± 21.2	45.3 ± 15.4	0.004
**Cobb angle of kyphosis (°)**	24.5 ± 20.0	48.2 ± 33.2	< 0.001	40.0 ± 34.7	33.1 ± 24.8	0.445	38.2 ± 33.2	33.8 ± 25.0	0.691
**AVT (mm)**	27.0 ± 11.1	42.7 ± 16.4	< 0.001	36.3 ± 18.9	33.5 ± 13.2	0.661	35.3 ± 15.7	34.2 ± 16.4	0.713
**TS (mm)**	12.8 ± 10.1	17.5 ± 12.4	0.007	15.8 ± 12.5	14.5 ± 10.7	0.646	15.1 ± 12.5	15.1 ± 10.3	0.621
**SVA (mm)**	28.8 ± 21.2	32.9 ± 22.3	0.212	29.5 ± 20.3	31.9 ± 23.0	0.731	31.8 ± 21.6	29.7 ± 22.1	0.469
**Morphology of HV (n & %)**									
**Width across CVL of DAV**	26 (32.9)	41 (53.2)	0.010	28 (40.0)	39 (45.3)	0.502	39 (45.9)	28 (39.4)	0.418
**Lateral height ratio**^**c**^ **≥ 0.9**	46 (58.2)	42 (54.5)	0.643	39 (55.7)	49 (57.0)	0.874	57 (67.1)	31 (43.7)	0.003
**Posterolateral position**	30 (38.0)	49 (63.6)	0.001	35 (50.0)	44 (51.2)	0.885	46 (54.1)	33 (46.5)	0.342
**Associated anomalies (n & %)**									
**Compensatory curve**	22 (27.8)	25 (32.5)	0.530	17 (24.3)	30 (34.9)	0.151	26 (30.6)	21 (29.6)	0.891
**Anteroposterior discordance**^**d**^	18 (22.8)	10 (13.0)	0.111	11 (15.7)	17 (19.8)	0.512	15 (17.6)	13 (18.3)	0.914
**Intraspinal anomalies**	19 (24.1)	17 (22.1)	0.770	17 (24.3)	19 (22.1)	0.746	18 (21.2)	18 (25.4)	0.538

Data are presented as mean ± standard deviation or number of patients (percentage)

*TS* trunk shift: *SVA* sagittal vertical axis: *AVT*: apex vertebral translation: *HV* hemivertebra: *CVL* central vertical line: *DAV* distal adjacent vertebra

^*^The raw *P* values are provided because there is no significant difference of the distribution of age between groups, and adjusting by age using analysis of covariance is unnecessary

^a^Intrinsic HV indicated that the total number of vertebrae including the HV was normal (equal to or less than 12 thoracic and 5 lumbar vertebrae) and the HV was numbered by the corresponding vertebral level, e.g. T12; Excess HV indicated that the HV was a supernumerary segment in addition to the 17 or more normally developed thoracic and lumbar vertebrae, and thus was numbered by the corresponding intervertebral space, e.g. T12-L1. ^c^Twice the convex lateral height of HV divided by the summation of convex lateral height of proximal and distal adjacent vertebra. ^d^The mismatch phenomenon between vertebral body and posterior structure of HV

**Fig. 4:**
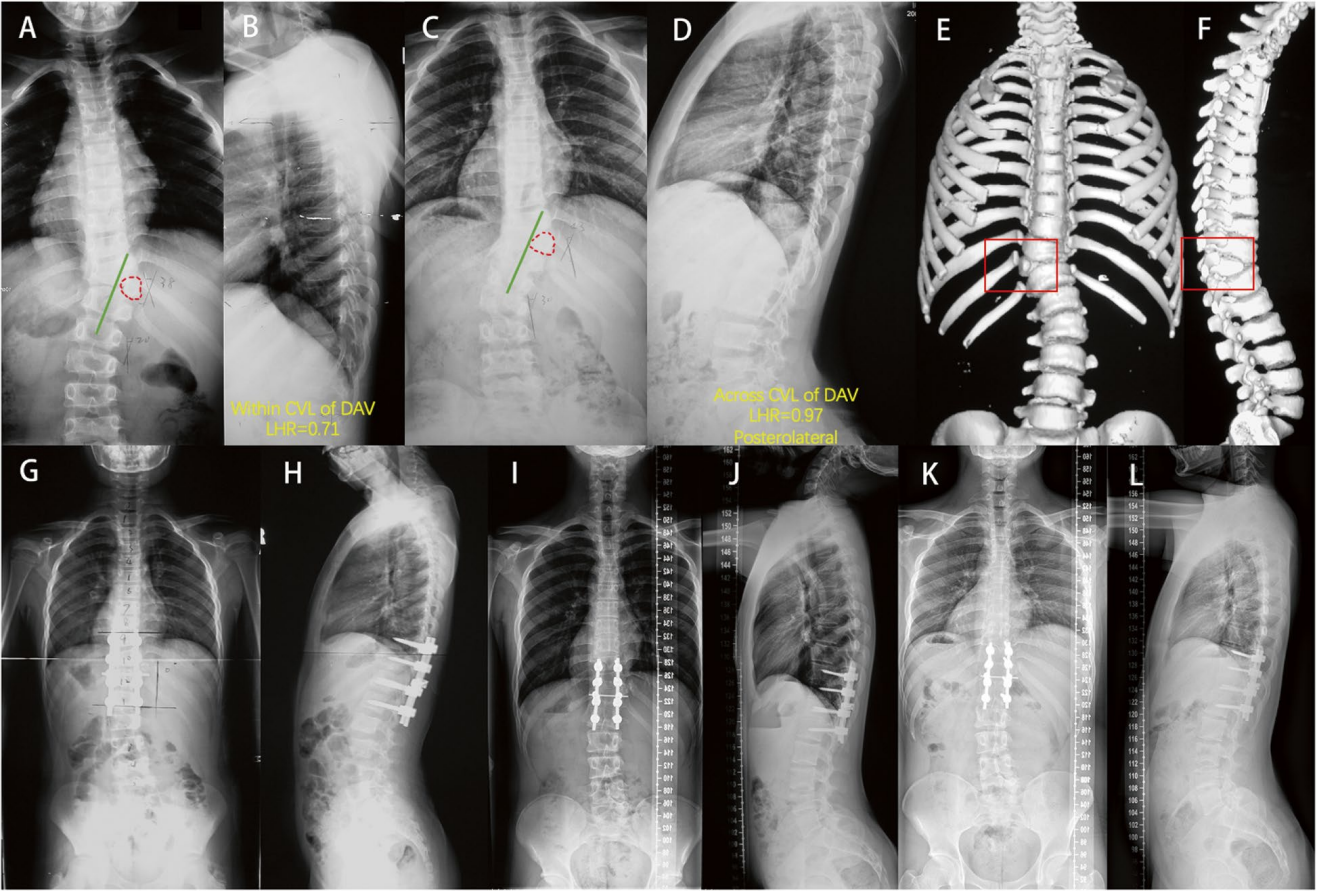
The images of a male patient who underwent posterior hemivertebra resection with short segment fusion at age 9. **A** & **B**, 2 years before operation; **C** to **F**, right before operation; **G** & **H**, immediately after operation; **I** & **J**, 4-year follow-up and **K** & **L**, 8-year follow-up. This patient underwent 2 years of observation before operation, during which the cobb angle of major and compensatory cure increased from 38° to 43° and from 20° to 30° respectively

**Fig. 5:**
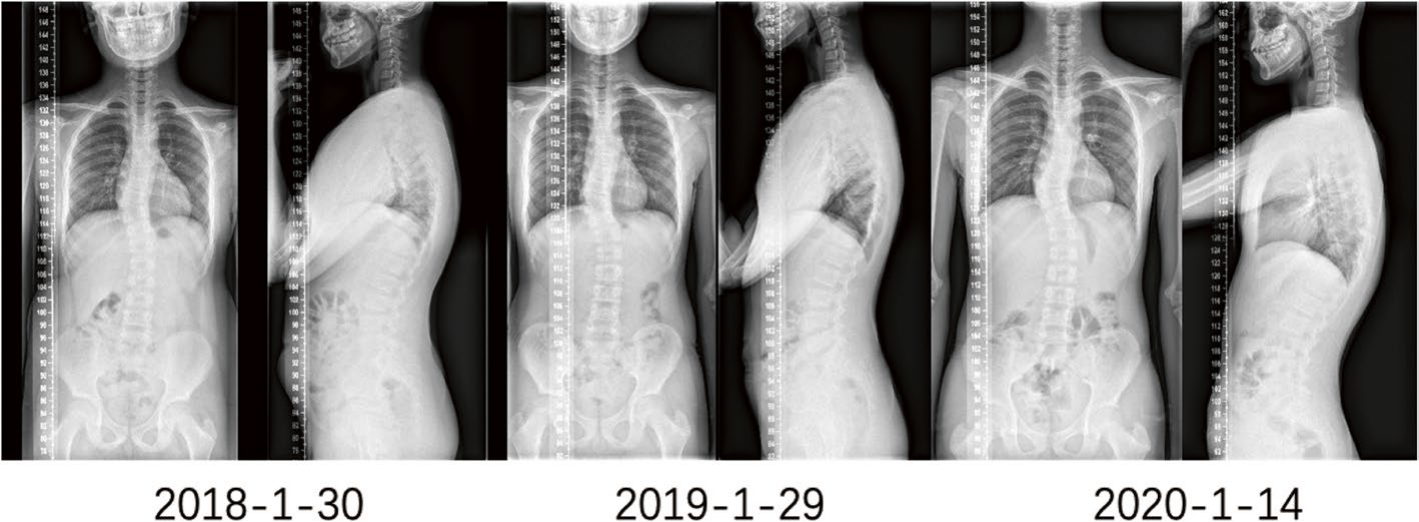
A patient chose conservative treatment and underwent three follow-up visits with a one-year interval, and X-ray images of the entire spine were displayed in both the anterior and lateral positions

Twenty-four patients (15.4%) were under clinical follow-up without surgery. In the other 132 surgically treated cases, 42 patients had an average of 2.1 ± 1.2 years of preoperative observation to verify the surgical indication (Fig. 4); the other 90 patients underwent operation immediately after initial visit due to the relatively severe deformity. Sufficient correction was achieved without HV resection in 12 patients (Fig. 5); HV resection was performed in the other 120 patients.

In the 156 isolated HVs, right-sided HV (55.1%) slightly outnumbered the left side (Fig. 3). The majority of HVs were located from T10 to L3–4 (80.1%, 125/156). The number of thoracic, thoracolumbar, and lumbar HVs were 63, 41, and 52, respectively. The three positive parameters of HV, i. e. HV across the midline, posterolateral HV and HV with an LHR ≥0.9 were identified in 67 (42.7%), 79 (50.3%) and 88 (56.1%) cases, respectively.

### The comparisons by the three morphological parameters of hemivertebra.

**Fig. 6:**
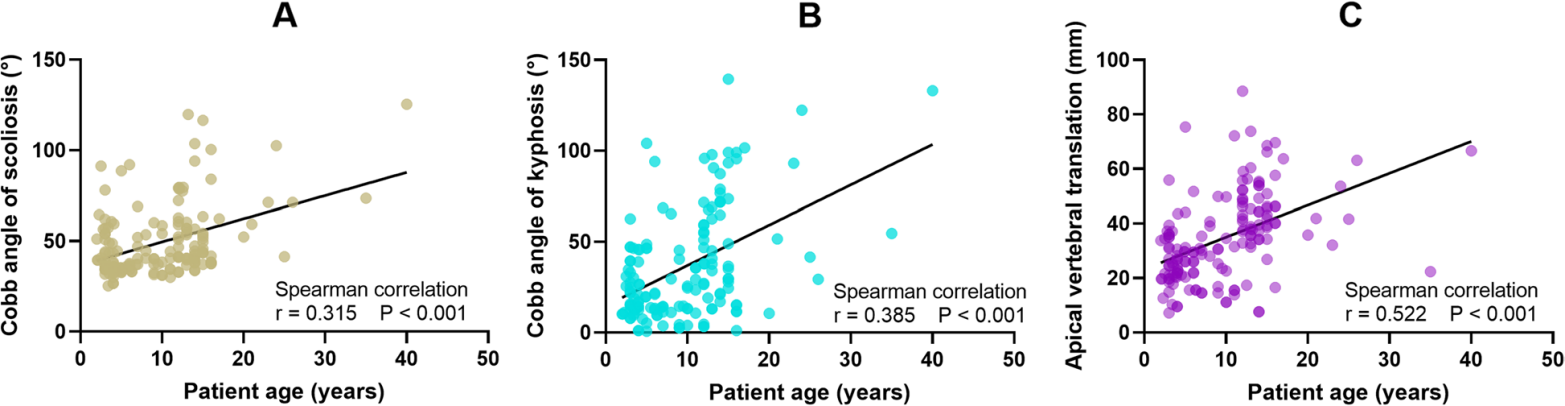
Patient age is positively correlated with scoliosis (**A**), kyphosis (**B**) and apical vertebral translation (**C**)

Patients who were 10 years old or older had significantly larger scoliosis, kyphosis, and AVT than patients under the age of 10 (*P* <  0.001, Table 1). Positive correlations between age and these three parameters of curvature were indicated by the Spearman coefficient (*P* <  0.001, Fig. 6). The correlation coefficients between patient age and scoliosis, kyphosis, AVT are 0.315, 0.385, and 0.522, respectively. The HV across midline and posterolateral HV were more common in older patients (53.2% vs. 32.9%, *P* = 0.010; 63.6% vs. 38.0%, *P* = 0.001, respectively). After adjusting by age using ANCOVA, HV across the midline had significantly larger scoliosis, kyphosis and AVT than HV within the midline (58.3 ± 20.6° vs. 42.8 ± 15.0°, *P* <  0.001; 45.1 ± 32.5° vs. 29.5 ± 25.7°, *P* = 0.013; 39.5 ± 17.7 mm vs. 31.2 ± 13.7 mm, *P* = 0.014, respectively; Table 2). The HVs with LHR ≥0.9 were only associated with larger Cobb angle of scoliosis (55.7 ± 20.6° vs. 41.4 ± 13.3°, *P* <  0.001). Significantly larger scoliosis, kyphosis, and AVT were observed in the posterolateral HVs (54.4 ± 21.0° vs. 44.4 ± 15.6°, *P* = 0.026; 51.4 ± 31.5° vs. 20.6 ± 17.1°, *P* <  0.001; 38.9 ± 16.9 mm vs. 30.5 ± 13.9 mm, *P* = 0.032; Table 2). Based on these findings, crossing the midline, posterolateral position and an LHR ≥0.9 can be summarized as the three positive parameters of HV that were associated with more severe deformity.

**Table 2:** The comparisons by the three morphological parameters of hemivertebra

Variables	Width of hemivertebra			Lateral height ratio^b^			Sagittal position		
	Within (*n* = 89)	Across (*n* = 67)	*P*-value (Adjusted)	< 0.9 (*n* = 68)	≥ 0.9 (*n* = 88)	*P*-value^#^	Lateral (*n* = 77)	Posterolateral (*n* = 79)	*P*-value (Adjusted)
**Patient age (years)**	9.0 ± 6.8	11.0 ± 5.9	0.015	9.7 ± 5.9	9.7 ± 6.5	0.651	8.1 ± 5.0	11.2 ± 6.9	0.003
**Deformity parameters**									
**Cobb angle of scoliosis (°)**^**a**^	42.8 ± 15.0	58.3 ± 20.6	< 0.001 (< 0.001)	41.4 ± 13.3	55.7 ± 20.6	< 0.001	44.4 ± 15.6	54.4 ± 21.0	< 0.001 (0.026)
**Cobb angle of kyphosis (°)**^**a**^	29.5 ± 25.7	45.1 ± 32.5	0.002 (0.013)	32.6 ± 27.5	39.0 ± 31.2	0.171	20.6 ± 17.1	51.4 ± 31.5	< 0.001 (< 0.001)
**AVT (mm)**^**a**^	31.2 ± 13.7	39.5 ± 17.7	0.002 (0.014)	33.9 ± 15.4	35.5 ± 16.5	0.503	30.5 ± 13.9	38.9 ± 16.9	0.001 (0.032)
**TS (mm)**	14.6 ± 9.8	15.7 ± 13.5	0.933	15.7 ± 11.1	14.6 ± 11.8	0.511	14.8 ± 9.8	15.4 ± 13.0	0.615
**SVA (mm)**	28.2 ± 20.6	34.4 ± 22.9	0.070	30.2 ± 23.0	31.4 ± 20.9	0.505	28.9 ± 20.9	32.8 ± 22.6	0.273
**Associated anomalies (n & %)**									
**Compensatory curve**	23 (25.8)	24 (35.8)	0.179	18 (26.5)	29 (33.0)	0.381	21 (27.3)	26 (32.9)	0.443
**Anteroposterior discordance**^**c**^	18 (20.2)	10 (14.9)	0.393	14 (20.6)	14 (15.9)	0.450	21 (27.3)	7 (8.9)	0.003
**Intraspinal anomalies**	19 (21.3)	17 (25.4)	0.555	12 (17.6)	24 (27.3)	0.157	21 (27.3)	15 (19.0)	0.219

Data are presented as mean ± standard deviation or number of patients (percentage).

*TS* trunk shift: *SVA* sagittal vertical axis: *AVT* apex vertebral translation.

^a^Adjusted by age using analysis of covariance (ANCOVA) when necessary. ^#^No significant difference of the distribution of age between two groups was identified, and adjusting by age using ANCOVA was unnecessary. ^b^Twice the convex lateral height of hemivertebra divided by the summation of convex lateral height of proximal and distal adjacent vertebra. ^c^The mismatch phenomenon between vertebral body and posterior structure of hemivertebra

### The influence of the location of hemivertebra on clinical manifestations in patients with congenital scoliosis caused by isolated hemivertebra.

When analyzed by the location of HVs, thoracolumbar HVs had the highest value in scoliosis, kyphosis, and AVT among three groups, of which the most significant statistical results occurred in kyphosis (*P* <  0.001, Table 3). Besides, the thoracolumbar group had a higher percentage of posterolateral HVs and lower incidence of APD than the thoracic and lumbar groups, with all the differences reaching a statistically significant level (*P* <  0.05, Table 3). The compensatory curve developed more frequently in patients with thoracic HV than the other two groups (52.4% vs. 19.5 and 11.5%, *P* = 0.002 and <  0.001, respectively, Table 3). Patients with ISA had a significantly higher proportion of APD than those without ISA (36.1% vs. 12.5%, *P* = 0.001).

**Table 3:** The influence of the location of hemivertebra on clinical manifestations in patients with congenital scoliosis caused by isolated hemivertebra

Variables	Region of HV			*P* value			
	Thoracic (T, T1 to T11–12, *n* = 63)	Thoracolumbar (TL, T12/13 to L1, *n* = 41)	Lumbar (L, L1–2 to L5/6-S1, *n* = 52)	Overall^*^	Post hoc test with Bonferroni correction		
					T vs.TL	T vs. L	TL vs. L
**Patient age (years)**	8.9 ± 5.7	11.8 ± 7.2	8.9 ± 5.7	0.065	N/A	N/A	N/A
**Male (n & %)**	38 (60.3)	21 (51.2)	22 (42.3)	0.156	N/A	N/A	N/A
**Left side HV (n & %)**	21 (33.3)	24 (58.5)	25 (48.1)	0.035	0.034	0.325	0.948
**Excess HV (n & %)**^**a**^	21 (33.3)	17 (41.5)	33 (63.5)	0.005	1.000	0.004	0.104
**Deformity parameters**							
**Cobb angle of scoliosis (°)**	50.3 ± 19.3	55.5 ± 23.1	43.6 ± 13.2	0.018	0.891	0.153	0.018
**Cobb angle of kyphosis (°)**	36.3 ± 24.1	55.1 ± 35.9	21.2 ± 17.2	< 0.001	0.073	0.001	< 0.001
**AVT (mm)**	32.6 ± 15.2	41.1 ± 18.5	32.4 ± 13.6	0.026	0.046	1.000	0.053
**TS (mm)**	11.8 ± 7.7	18.1 ± 14.8	16.6 ± 11.7	0.059	N/A	N/A	N/A
**SVA (mm)**	30.9 ± 22.5	39.3 ± 23.3	24.0 ± 17.1	0.004	0.128	0.373	0.003
**Morphology of HV (n & %)**							
**Width across CVL of DAV**	30 (47.6)	21 (51.2)	16 (30.8)	0.088	N/A	N/A	N/A
**Lateral height ratio**^**b**^ **≥ 0.9**	45 (71.4)	22 (53.7)	21 (40.4)	0.003	0.193	0.002	0.607
**Posterolateral position**	31 (49.2)	31 (75.6)	17 (32.7)	< 0.001	0.022	0.222	< 0.001
**Associated anomalies (n & %)**							
**Compensatory curve**	33 (52.4)	8 (19.5)	6 (11.5)	< 0.001	0.002	< 0.001	0.857
**Anteroposterior discordance**^**c**^	11 (17.5)	0 (0)	17 (32.7)	< 0.001	0.014	0.175	< 0.001
**Intraspinal anomalies**	16 (25.4)	7 (17.1)	13 (25.0)	0.568	N/A	N/A	N/A

Data are presented as mean ± standard deviation or number of patients (percentage).

*TS* trunk shift: *SVA* sagittal vertical axis: *AVT* apex vertebral translation: *HV* hemivertebra: *CVL* central vertical line: *DAV* distal adjacent vertebra: *N/A* not applicable.

^*^The raw *P* value is provided because there is no significant difference of the distribution of age between groups and adjusting by age using analysis of covariance is unnecessary.

^a^Excess HV indicated that the HV was a supernumerary segment in addition to the 17 or more normally developed thoracic and lumbar vertebrae, and thus was numbered by the corresponding intervertebral space, e.g. T12-L1. ^b^Twice the convex lateral height of HV divided by the summation of convex lateral height of proximal and distal adjacent vertebra. ^c^The mismatch phenomenon between vertebral body and posterior structure of HV

### The outpatient follow-up data of 66 patients with isolated hemivertebra.

The follow-up data were available in 66 patients. These patients were classified based on width of hemivertebra, Lateral height ratio and Number of the positive parameters, with no statistically significant difference in the corresponding age of diagnosis. Since their diagnosis, these patients undergo regular follow-up to assess disease progression, with a follow-up period of approximately 2 years. The patients who received clinical observation followed by surgery had higher annual progression of scoliosis than those who required observation only (4.7 ± 1.9° vs. 2.7 ± 0.8°, *P* <  0.001). After comparing the initial visit and the latest follow-up, we can calculate the total progression and take the average value to calculate the annual progression. In comparisons by each positive parameter, HVs across the midline (*n* = 19) and HVs with an LHR ≥0.9 (*n* = 33) had higher annual progression of scoliosis (5.2 ± 2.4° vs. 3.4 ± 1.3°, *P* = 0.004; 4.5 ± 2.0° vs. 3.4 ± 1.5°, *P* = 0.005, respectively). No statistical difference in progression was identified between lateral and posterolateral HVs. When analyzed by the number of existing positive parameters, HVs with two to three positive parameters had significantly higher annual progression than HVs with zero to one positive parameter (5.0 ± 2.2° vs. 3.3 ± 1.3°, *P* <  0.001; Table 4). As the number of positive parameters rose from zero to three, the proportion of the patients who required surgery increased significantly (*P* <  0.001; Table 5).

**Table 4:** The outpatient follow-up data of 66 patients with isolated hemivertebra

Variables	Width of hemivertebra			Lateral height ratio^a^			Number of the positive parameters^b^		
	Within (*n* = 47)	Across (*n* = 19)	*P* value^#^	< 0.9 (*n* = 33)	≥ 0.9 (*n* = 33)	*P* value^#^	0 to 1 (*n* = 42)	2 to 3 (*n* = 24)	*P* value^#^
**Age at diagnosis (years)**	6.1 ± 4.2	6.5 ± 3.9	0.859	7.1 ± 4.0	5.3 ± 4.1	0.087	6.7 ± 4.0	5.3 ± 4.2	0.135
**Duration of follow-up (years)**	2.3 ± 1.4	2.0 ± 1.3	0.332	2.3 ± 1.4	2.2 ± 1.3	0.802	2.6 ± 1.4	1.8 ± 1.2	0.070
**Cobb angle of scoliosis (°)**									
**Initial visit**	32.3 ± 8.8	43.2 ± 13.9	0.002	32.7 ± 7.3	38.3 ± 14.2	0.138	33.5 ± 8.6	39.0 ± 15.0	0.176
**The latest follow-up**	39.9 ± 10.5	52.1 ± 16.9	0.001	39.4 ± 7.5	47.4 ± 17.1	0.135	40.8 ± 10.8	48.1 ± 17.0	0.088
**Total progression**	7.6 ± 4.3	8.9 ± 5.9	0.272	6.7 ± 3.1	9.2 ± 5.8	0.058	7.3 ± 4.4	9.1 ± 5.3	0.045
**Annual progression**	3.4 ± 1.3	5.2 ± 2.4	0.004	3.4 ± 1.5	4.5 ± 2.0	0.005	3.3 ± 1.3	5.0 ± 2.2	< 0.001

Data are presented as mean ± standard deviation or number of patients (percentage).

^a^Twice the convex lateral height of hemivertebra divided by the summation of the convex lateral height of proximal and distal adjacent vertebra. ^b^Comprising the three morphological parameters of hemivertebra: width across the midline, b lateral height ratio ≥ 0.9 and posterolateral position.

^#^Calculated with Student’s t-test and Mann-Whitney U test as appropriate

**Table 5:** Distribution of parameters in four groups of patients with isolated hemivertebra

Variables	Number of the positive parameters^a^				*P* value
	0 (*n* = 12)	1 (*n* = 30)	2 (*n* = 17)	3 (*n* = 7)	
**Age at diagnosis (years)**	5.5 ± 4.0	7.2 ± 4.0	4.5 ± 4.0	7.6 ± 4.1	0.081
**Location of hemivertebra**					0.132
**Thoracolumbar (T12/13-L1)**	2 (16.7)	5 (16.7)	4 (23.5)	4 (57.1)	
**Non-thoracolumbar**	10 (83.3)	25 (83.3)	13 (76.5)	3 (42.9)	
**Treatment**					< 0.001^#^
**Observation (n & %)**	12 (100)	10 (33.3)	2 (11.8)	0 (0)	
**Observation followed by surgery (n & %)**	0 (0)	20 (66.7)	15 (88.2)	7 (100)	

Data are presented as number of patients (percentage).

^a^Comprising the three morphological parameters of hemivertebra: width across the midline, a lateral height ratio ≥ 0.9 and posterolateral position.

^#^Calculated by the Mantel-Haenszel chi-square test. In the post hoc tests with Bonferroni correction, group 1, 2 and 3 had significantly higher percentage of patients who required surgery than group 0 (*P* = 0.001, < 0.001, and < 0.001, respectively); no intergroup difference was identified between group 1, 2 and 3 (*P* > 0.05)

## Discussion

This study is a small sample, single center study aimed at improving the measurement and evaluation of the hemivertebra. Although there are selection bias, information bias, and confounding bias, they have significant implications for clinical diagnosis and treatment. Previous research has focused on natural history studies related to CS and neglected in-depth research on HV. This study improves the relevant definitions and conducts quantitative analysis to systematically study the relationship between HV morphological parameters and the severity of malformations. The limitations of this study were the relatively small proportion of outpatient follow-up data and the single-center retrospective design. The fact that patients with severe disease are transferred to our hospital also prohibited us from collecting more mild-to-moderate cases. Future studies with multicenter cohorts and complete follow-up data will better reveal the relationship between the morphology of HV and the course of CS.

The severity and progression of CS caused by HV varies greatly and is difficult to predict [1, 2, 4–6, 16]. Accordingly, the principle of treatment is controversial from early HV resection to observation [3]. It is important to assess the probability of progression in isolated HV to justify the treatment strategy. In real-world clinical practice, the majority of CS patients underwent operations immediately after the initial visit due to the severity of deformity and thus, the longitudinal data were unavailable. As a workaround, the present study analyzed the data from both cross-sectional evaluation and outpatient follow-up to identify possible relevant factors of curve progression.

The present study scrutinized the 3-D morphology of HV by three geometrical parameters, transverse width, LHR, and sagittal position, which were briefly mentioned in the literature [1, 4]. The width of the HV represents the medial growth potential, and the lateral height reflects the longitudinal growth. The sagittal position is the combined effect of the anterior dysplasia and rotation of vertebral body. The three positive parameters of HV, width across the midline, posterolateral position, and LHR ≥0.9, were all associated with more severe deformity. According to the follow-up data, two of these three parameters, width across the midline and LHR ≥0.9 also indicated a faster progression of scoliosis. The number of positive parameters in an HV was analyzed, for it can overcome the limitation of a single parameter. The existence of more than one positive parameter of HV may serve as an indicator of progression.

In this study, the patients’ age peaked under 5 years old and around puberty, which was similar to previous studies [2, 6], and corresponded with two rapid growth periods of the spine [24]. Due to the growth of HV, older patients were associated with significantly more severe deformity, which was consistent with related studies [4, 6]. This finding suggested that early diagnosis of HV-induced CS is of great importance. Another finding is that the deformity was the most severe in the thoracolumbar HVs (T12-L1) than in the thoracic and lumbar HVs [1, 2, 4, 6]. Therefore, closer observation and more aggressive treatment are needed in thoracolumbar HVs.

Based on the above results, a preliminary scheme of treatment can be drawn up according to the number of positive parameters in an HV. In patients with CS caused by isolated HV, prophylactic HV resection may be necessary for HVs with more than one positive parameters, given that 91.7% (22/24) of the patients who underwent observation at first eventually required surgery. On the other hand, if the deformity is mild and no more than one of the aforementioned three positive parameters is identified, then biannual observation is recommended.

The existing research on HV mainly focuses on the description of the anterior column and is mostly based on two-dimensional images on X-ray plain films, with little mention of the posterior structure. A typical HV has a semi vertebral plate corresponding to the anterior column, but like the anterior vertebral body, the size, shape, and segmentation of the posterior vertebral plate can vary in various ways. A fully segmented HV in the front can correspond to a fully segmented semi vertebral plate, semi segmented vertebral plate, or bilateral vertebral plate. Poor segmentation of the posterior vertebral plate may also affect the progression of CS. Only by fully understanding both the anterior and posterior structures can we comprehensively analyze the morphological characteristics of HV and make relatively accurate judgments on prognosis. Due to overlapping projection images, ordinary X-rays can only display the anterior vertebral body, and their ability to distinguish the posterior structure is relatively limited. In cases with severe kyphosis or extensive complex vertebral deformities, X-ray imaging of the anterior vertebral body is also limited. Although the posterior structure can be observed under direct vision during surgery, the exact correspondence between the anterior vertebral body and the posterior structure is still unknown. When locating the HV segment, experience and repeated intraoperative X-ray fluoroscopy are necessary. The development of CT and 3D reconstruction technology enables researchers to observe various structures of the spine from multiple perspectives in a more intuitive way, which is a powerful means to reveal the relationship between HV and adjacent vertebral bodies, as well as the corresponding relationship between anterior and posterior structures.

In conclusion, the present study identified three positive parameters of HV that were associated with a more severe deformity in CS caused by isolated HV: width across the midline, posterolateral position, and LHR ≥0.9. Selective observation is recommended for cases with mild deformity when none or one of the three parameters is identified. Prompt resection is rational for HVs with more than one positive parameter.

## Data Availability

The datasets generated during and/or analysed during the current study are available from the corresponding author on reasonable request.

## Abbreviations

ANCOVA: Analysis of covariance
APD: Anteroposterior discordance
AVT: Apical vertebral translation
CS: Congenital scoliosis
CT: Computed tomography
CVL: Central vertical line
DAV: Distal adjacent vertebra
ESA: Extraspinal anomalies
HV: Hemivertebra
ISA: Intraspinal anomalies
LHR: Lateral height ratio
MR: Magnetic resonance imaging
SVA: Sagittal vertical axis
TS: Trunk shift
